# Abdominal Pain in Childhood
*Being the substance of a lecture delivered before the Medico-Chirurgical Society of Bristol on May 13th, 1936.


**Published:** 1936

**Authors:** Wilfrid Sheldon

**Affiliations:** Physician for Diseases of Children, King's College Hospital; Physician to Out-patients, Hospital for Sick Children, Great Ormond Street


### ABDOMINAL PAIN IN CHILDHOOD.*

BY

WILFRID SHELDON, M.D., F.R.C.P.,

*Physician for Diseases of Children, King's College Hospital; Physician to Out-patients, Hospital for Sick Children, Great Ormond Street.*

---

It is eminently desirable that a lecturer who has the honour of addressing the Medico-Chirurgical Society should select a subject of interest to both surgeons and physicians, and for this reason I have chosen abdominal pain, for there can hardly be a clinical symptom more evenly shared by medicine and surgery. In childhood the subject is made more difficult by the inability of the patient to describe his pains or even to localize them with precision, and sometimes, too, by a disinclination to submit to examination. The difficulties are perhaps greatest in infancy, when one has to rely for the history on the parents' statements.

### PAIN IN INFANCY.

A full and complete history is the first essential, and the time spent on eliciting the various points and marshalling them in their proper sequence should never be grudged. An infant naturally reacts to pain by crying, and much help is to be obtained

* Being the substance of a lecture delivered before the Medico-Chirurgical Society of Bristol on May 13th, 1936.

from the characters of the cry. Needless to say, pain is not the only cause of crying—hunger, cold, a soiled napkin, or even boredom are more frequent reasons, but if the infant is carefully observed, it is often possible to tell when the crying is due to pain.

The features which should always be noted are whether the crying is continuous or intermittent, whether it is a short, sharp scream or a piteous wailing, whether it occurs at any particular time of the day, and whether it is related to meal-times or to such physiological acts as sucking, defæcation or micturition. The importance of these points may be illustrated by recalling the intermittent pains of an acute intussusception, the sharp screams that accompany meningitis or acute otitis media (the latter especially at night time), the interference with sucking caused by thrush, and the scream that precedes micturition when the tip of the glans penis is ulcerated.

The attitude of the infant during the attack of pain should also be noted. Intestinal colic from any cause is likely to make the infant double up, with the thighs flexed on the abdomen and the arms pressed into the sides; banging and rolling the head often accompany cephalic pain; while the bouts of renal pain that occur in acute pyelitis, and from other urinary causes, may make the infant lie rigid with arched back.

### EXAMINATION OF THE ABDOMEN.

The physical examination of the abdomen is made more valuable by paying attention to the small technical details. For instance, in infancy the napkin covers so much of the abdomen that an examination without the napkin removed is a waste of time, and also at this age it is advantageous to examine while the infant is being fed, for then the baby is quiet and lies still, and the abdomen is relaxed and easy to feel. The examining hand should always be warm, and the examiner should be on the same level as the child. Palpation should begin in the quadrant farthest from the area under suspicion, leaving the painful area until the last. Valuable information may come to light if the child's face is watched while the abdomen is being palpated, for when a tender area is reached, whether it be over an acute intussusception, over an inflamed appendix, or in the loin over a kidney the seat of pyelitis, the child may be seen to start or wince. A point that is perhaps worth mentioning is that when an infant is very wasted, or suffering from a severe loss of fluid as in acute gastro-enteritis, the abdominal wall becomes so toneless that the outline of the underlying intestine may easily be made out, and the bowel can be seen undergoing peristalsis, but this should not be interpreted as indicating intestinal obstruction. Lastly, the value of a rectal examination is as great in childhood as at any other age, and there need be no hesitation in making the examination even in infancy, for the rectum of a newborn child is quite capable of accommodating an adult forefinger. The finger must, of course, be inserted slowly, using no force, but keeping the finger-tip lightly pressed against the anus until it is slowly engulfed.

### PAIN ARISING OUTSIDE THE ABDOMEN.

Abdominal pain is so common a complaint in children that unless causes arising outside the abdomen are first of all excluded as a routine serious mistakes may easily arise. One of the most important is tuberculous caries of the spine, which in its incipient stage may give rise only to abdominal pain. The pain is referred along the intercostal nerves to the anterior abdominal wall, and theoretically it should be possible to tell that the pain is superficial, without deep tenderness, but in practice this is difficult to appreciate in children. It is, however, possible to make a habit of examining the child's back and noting whether there is any stiffness when the spine is bent this way and that, for stiffness is present for some time before the characteristic kyphosis appears. Another difficulty which often arises is in deciding whether abdominal pain is due to appendicitis or to an early pneumonia. The pain in pneumonia is actually due to an accompanying pleurisy, and as the pleura is much more likely to be involved in lobar pneumonia than bronchopneumonia the differential diagnosis is generally between appendicitis and the lobar variety. In general the temperature at the onset of pneumonia is higher than at the onset of appendicitis ; flushing of the cheeks may be present, and the respiration-rate may rise out of proportion to the increase in pulse-rate. When the chest is examined the most important early sign of pneumonia is a diminished entry of air over the affected lobe of a lung, for this precedes by several hours or sometimes by days the better-known signs of an impaired percussion note and tubular breathing. If, after a careful examination, a doubt is still entertained, it is better to defer the diagnosis for a few hours, by which time the clinical picture will have become more definite.

Another disease which may be deceptive is rheumatism. Occasionally there is superficial pain in the muscles and aponeuroses of the abdominal wall comparable with the vague aches and pains that occur in the limbs, and which are usually attributed to rheumatism, although it may be added that the evidence that they bear any relation to the more severe forms of rheumatism such as carditis or arthritis is meagre. Greater difficulty arises when acute rheumatic arthritis (rheumatic fever) is confined to one joint, for should the right hip-joint be affected there is likely to be pain and rigidity referred to the right lower quadrant of the abdomen, thus simulating appendicitis. In both conditions the hip-joint is likely to be held flexed, but while movement of the rheumatic joint is exquisitely painful, this is not the case with appendicitis. Severe abdominal pain may also be caused by rheumatic pericarditis, but the accompanying signs of an enlarged and tumultuous heart, and also the presence of bruits and perhaps friction sounds, should prevent error. Another condition which may be misleading unless a complete examination is carried out is acute tonsillitis, for this may sometimes be accompanied by pain and tenderness over the area of the appendix. The most probable explanation would seem to be that the child swallows infected mucopus from the nasopharynx, which sets up a mild enteritis, with some actual inflammation of the appendix. The abdominal symptoms subside, however, as soon as the throat infection settles down, and operation is not called for, although it may be undertaken in error unless a routine examination of the throat is made in all children in whom appendicitis is suspected.

Other conditions which must be mentioned for the sake of completeness are leukæmia, which gives rise to abdominal pain probably as the result of acute swelling of the deep abdominal lymphatic glands; herpes zoster, which may cause acute superficial pain over the area where twenty-four hours or so later the typical rash is to appear; and diabetic coma, the onset of which in childhood is likely to be marked by such severe vomiting, abdominal pain, and even a degree of rigidity as to resemble some form of intestinal obstruction. The presence of sugar and ketone bodies in the urine will, however, indicate the correct diagnosis, and therefore it is essential that a specimen of urine be obtained before accepting a diagnosis of intestinal obstruction.

Lastly, one may mention the pain which accompanies inguinal hernia. Infants with an inguinal rupture are almost always poorly nourished, and their crying and general behaviour suggests that they are actually in pain. Of course, it may be that the abdominal strain that accompanies crying has originally caused the rupture, but it has often seemed to me that a hernia forced down into the scrotum causes the infant a good deal of pain.

### PAIN REFERRED FROM THE ABDOMEN.

The only variety at all common in childhood is pain referred to the tip of the shoulder as a result of inflammation in the neighbourhood of the diaphragm. As a rule it is due to diaphragmatic pleurisy, but may occasionally be caused by pericarditis. Subphrenic inflammation with pain at the tip of the right shoulder is most likely to be due to a subphrenic abscess arising from appendicitis, while pain at the tip of the left shoulder may be caused by perisplenitis, and may also be met with when the spleen is ruptured. Pain with this localization following an accident should always put one on one's guard lest the spleen be injured.

### ACUTE ABDOMINAL COLIC.

The most common cause of acute colic is some indiscretion in diet. There may be a history of the child having eaten indigestible material, and almost always after a few hours the passage of loose undigested motions leads to the right diagnosis. So long as the diagnosis is uncertain an expectant attitude must be taken. This means that a purgative should not be ordered, but relief may be given by a warm enema and by fomentations.

Another important cause of colicky pain is acute intussusception. The usual history is that a young child, previously in splendid health, suddenly has an acute attack of pain. The pain recurs at intervals of a few minutes, each one being severe enough to make the child cry out and double himself up. The passage of a little blood and mucus in the stools usually occurs after a few hours. The point to be stressed is that the pain is intermittent and severe. Careful palpation of the abdomen may reveal the typical sausage-shaped tumour, but this may be difficult to feel when the abdomen is tense and the child is crying ; even so, if the child's face is closely watched it may be clear that there is tenderness at some point along the line of the colon. A rectal examination may bring away a little blood on the finger-stall and the intussusception can occasionally be felt per rectum. If there is a doubt a barium enema may be given, for the barium when it reaches the intussusception tracks along as a thin line between the two layers of bowel, giving a typical picture.

Occasionally the intussusception becomes subacute or chronic. In that case the blood supply to the intussuscepted portion of bowel is not cut off, the bowel remains alive, and small stools may continue to be passed. The child may not be brought for examination until two or three weeks have passed, but if a careful history is taken the onset will be found to have been exactly like an acute intussusception, while since then colicky attacks of pain have occurred from time to time and there has been a steady loss of weight. A chronic intussusception is so uncommon that the two characteristic physical signs should be elicited before the diagnosis is accepted ; these consist of a palpable tumour which undergoes alternate hardening and softening, and waves of peristalsis which sweep along the colon to fade away when the tumour is reached.

Henoch's purpura is one of the conditions from which an acute intussusception has to be distinguished. It is, however, uncommon. The abdominal features consist of recurrent colicky pains and the passage of blood and mucus in the stools, but as a rule no tumour can be felt, and a complete examination will generally show some purpuric spots in the skin or inside the mouth. Another usual feature of Henoch's purpura is œdema, occurring in patches and often situated in some bizarre site such as the forehead or on the chest. It may precede the appearance of purpura by a day or so, and therefore when a child is seen on account of a patchy œdema of this sort the possibility of an oncoming purpura should be borne in mind.

Acute appendicitis is seldom met with under three years of age. When taking the history the exact sequence of events is most important, for with appendicitis pain is always the initial symptom. Vomiting may be slight or even absent, but even when it occurs it never precedes the pain. On the other hand, it is usual in cyclic vomiting for the pain to occur some few hours after the vomiting. The pain of acute appendicitis takes two forms. At first the child complains of colicky pain in the region of the umbilicus. There is as yet no rigidity, and it is in this stage that the condition may easily be mistaken for indigestion, and the mistake be made of giving a purgative. After a few hours the pain changes its character: it becomes more sharp and stabbing, and the child may now be able to localize it to the right iliac fossa. The abdominal wall over the inflamed appendix becomes tender and rigid, and the diagnosis at this stage is usually straightforward enough. On the other hand, the appendix is occasionally out of its usual position, and the physical signs will vary accordingly. If the inflamed organ stretches down into the pelvis and lies between the bladder and rectum the pain and tenderness will be over the hypogastrium, and there are likely to be sudden uncontrolled evacuations of urine and fæces. In other cases the appendix lies behind the ascending colon, and there may be then no rigidity of the anterior abdominal wall, but tenderness can be elicited low down in the right loin. When a mild attack of appendicitis passes off without recourse to operation the pain gradually subsides, but a *sudden* disappearance of pain may be calamitous, for it is likely to occur when the appendix ruptures into the peritoneum. The rigidity of the abdominal wall increases and becomes more diffuse, and after a few hours the pulse rate rises. In such a case the need for operation is urgent, and until it can be undertaken the child must be kept absolutely at rest.

The occurrence of abdominal pain with acute tonsillitis has already been mentioned. In some children acute infections in any part of the body are likely to be accompanied by high temperature, repeated vomiting, abdominal pain, and drowsiness due to ketosis. Almost always the history shows that the vomiting has preceded the abdominal pain, the latter being probably due to the straining of the abdominal wall as a result of the incessant retching. These are features which apply also to attacks of cyclic vomiting, indeed it is probable that many cases termed "cyclic vomiting" are in reality due to ketosis accompanying recurrent infections in the throat or elsewhere. The chief difference is that cyclic vomiting is a truly periodic condition, the attacks following each other at regular intervals, while of course recurrent infections occur at quite irregular intervals.

Symptoms of acute intestinal obstruction sometimes arise in association with deformities of the mesentery. The most common deformity is one in which the mesentery of the small intestine is continued as a mesentery of the large bowel, and at the same time is attached to the posterior abdominal wall only at its upper end, that is, on the left side of the second lumbar vertebra. The result is that the whole intestine is much more movable than it should be, and kinks and twists are likely to arise. The symptoms of this condition have been pointed out by Waugh. Attacks of pain, vomiting, and constipation occur, and may simulate appendicitis, while in addition there may be a noticeable fullness in one or other quadrant of the abdomen, where for the time being the intestines are bunched together. These are the cases in which, if an operation for appendicitis is undertaken, the appendix may be found in some unusual quarter of the abdomen. The attacks are likely to be repeated, and may thus be easily mistaken for cyclic vomiting. Abnormalities of the mesentery are more common than might be supposed, and they are most likely to be fatal in the early months. Thus in a series of post-mortems at Great Ormond Street mesenteric deformities accounted for six deaths, all being young infants under six months of age ; during the period that they came to autopsy 176 infants under six months of age died from other causes, giving a post-mortem incidence in that particular age group of 3·5 per cent.

One other condition that must be mentioned is pneumococcal peritonitis. This is uncommon, and tends to occur more often in girls, in whom the peritoneum is usually infected as the result of an ascending infection via the genital tract. Peritonitis begins in the pelvis, and the pain and tenderness are therefore first felt in the hypogastrium. On examination the rigidity is also at first confined to the lower half of the abdomen. Vomiting generally occurs, and it is to be noted that diarrhœa, which is quite an exceptional feature in other forms of peritonitis, is usual in pneumococcal peritonitis, being accounted for presumably by the irritation of the lower bowel.

### CHRONIC ABDOMINAL PAIN.

*Chronic Indigestion.*—Pain of a rather indefinite niggling character is a common complaint in children of all ages, and is often associated with other evidence of a disordered digestion. Thus it is likely to be associated with a failure to thrive, with disturbances of appetite, and with the passage of unhealthy motions, which are often loose and offensive and may contain a good deal of mucus. The child tires easily, and the stance may at once suggest chronic fatigue, the chest being flattened, the abdomen protuberant, the shoulders rounded, the scapulæ projecting, and the chin sunken ; the complexion is sallow, the eyes are darkly ringed, the tongue may show a patchy distribution of fur, and postural or functional albuminuria may be present. Not uncommonly these symptoms may be combined with constipation, indeed constipation is sometimes the underlying cause. Other cases are associated with evidence of chronic infection of the tonsils and adenoids, the abdominal symptoms being due to an intestinal catarrh brought on by the repeated swallowing of mucopus from the nasopharynx. Dental caries is sometimes a factor, for without the wherewithal for efficient mastication the child tends to bolt his food, and this eventually spoils the digestion. Bad home management in the direction of irregular meal-times and unsuitable or indigestible food is sometimes the starting-point. Whatever the underlying factor may be, there is no doubt that these cases are common, and in the minds of both parents and doctors the symptoms often suggest that the child may be suffering from some form of intra-abdominal tuberculosis, although the long history and a careful analysis of the symptoms and signs will help to exclude such an infection. The management of these cases is always a prolonged affair; the diet needs to be an easily digestible one, from which all coarse foods and roughage are excluded; raw fruit, nuts, root vegetables, peas and beans, and coarse cereal foods are among the articles of diet which should be omitted for a few months. A mildly laxative alkaline bitter mixture before meals, such as a combination of rhubarb, soda, and infusion of gentian, is helpful; massage, regulated exercises and adequate rest periods each day will also be required if the child is to make a proper recovery.

Another common difficulty is to decide whether repeated attacks of abdominal pain are to be attributed to recurrent inflammation of the appendix. That recurrent appendicitis occurs cannot be doubted, but it is certainly much less common than chronic intestinal indigestion. Almost always it is associated with a history of constipation, although whether the latter is a causal factor is difficult to say. The diagnosis should not be entertained unless there is a clear history that in one or more of the attacks of pain there has been definite tenderness in the appendix area. Opinions differ as to the value of X-ray investigation in these cases; my own view is that provided that there is a history of tenderness over the appendix during attacks corroborative evidence sufficient to justify an exploratory operation is afforded if the appendix fills with barium, but fails to empty for twenty-four hours or more after the rest of the bowel is clear. Less value attaches to tenderness elicited over the appendix when the child is examined under the X-ray screen, for the child is in so strange an environment that little value can be attached to his sensations, and if the right iliac fossa is palpated with a hand encased in an X-ray-proof glove it would indeed be surprising if the child did not exhibit tenderness.

The question of abdominal tuberculosis has already been mentioned, and it is one which often arises. Tuberculous ulceration of the intestine gives rise to such rapid loss of flesh, diffuse abdominal tenderness and tumidity, and fever, with loose bowel actions sometimes containing blood, that it is not likely to be overlooked; while the physical signs of ascites or of a packed doughy abdomen seldom leave room for doubt about tuberculous peritonitis. The diagnosis is more difficult when the mesenteric glands only are involved. The infection reaches the glands by penetrating the lower part of the small intestine and the region of the cæcum, and therefore the glands first affected are generally those at the lower end of the mesentery in the right iliac fossa. The first essential in the diagnosis of tabes mesenterica is that the enlarged glands should be palpable. It is often difficult to tell whether the small masses in this area are actually glands or fæces; fæces are likely to be more movable, and are not as a rule tender, but even so the first examination is very likely to leave a doubt, in which case it is better to defer the final diagnosis until the effect of a course of laxatives over a week or so has been observed. Not infrequently at the end of this time masses which were thought to be glands will have moved on. Other evidence of tabes mesenterica is usually vague ; pain may be complained of and may be colicky in character, but more often amounts merely to a vague discomfort which the child is unable to localize. Nutrition is interfered with to the extent that the child ceases to put on weight, but actual wasting does not usually occur. Investigations, such as tuberculin tests and X-ray examination, may sometimes give corroborative evidence, but their interpretation needs caution. Thus a positive tuberculin skin test after three years of age may merely indicate that at some time in the past the child has been infected with tuberculosis from which he has recovered, and it does not follow that his abdominal symptoms are due to tuberculosis ; an X-ray of the abdomen will show tuberculous glands if healing by calcification has already commenced, but if the infection is recent the X-ray film is not likely to show anything abnormal. Without doubt, the most important point in the diagnosis is that the glands should be actually palpated.

It sometimes happens that the intestine becomes adherent to tuberculous glands, and even years after the glands have healed the adhesions may give rise to periodic kinking or twisting of the bowel with symptoms of partial obstruction. There is then a history that the child has periodic bouts of severe vomiting accompanied by constipation, and preceded by abdominal distension which subsides during the attack.

### Urinary Pain.

Pain arising from the urinary tract is more common in childhood than is often supposed. One cause which is easily overlooked is ulceration at the external urinary meatus of the penis. Almost always the child has been circumcised, and the history is that the infant screams when he passes his water, and the first drops of urine may be stained with blood. The pain is due to exudate from the ulcer sealing over the urinary meatus, and this seal has to be burst open before the urine can escape. The ulcer is often so minute that a close inspection is needed to see it. Treatment consists of making the urine mildly alkaline and keeping the end of the penis freely smeared with a protective layer of vaseline.

Acute pain, severe enough to simulate renal colic, may sometimes accompany acute pyelonephritis. During the attack the child screams and may be sick, and afterwards is left pale and exhausted. The pain is probably due to the passage of clots of pus down the ureter. Another group of conditions which may cause severe attacks of pain and vomiting are the various congenital deformities of the urinary tract which lead to urinary stasis, and eventually give rise to an intermittent hydronephrosis. The history is likely to be one of recurrent " biliousness," each attack lasting for two or three days and being accompanied by diffuse abdominal pain and vomiting. These children may easily be treated for cyclic vomiting, or regarded as instances of recurrent appendicitis, until examination actually during an attack fails to bring to light any good evidence of intestinal disturbance, but may perhaps reveal the presence of an enlarged kidney. Sooner or later the deformed urinary tract becomes infected, usually with the bacillus coli, and then in addition to the previous symptoms the child becomes pale, loses weight, the temperature is raised, perhaps over a degree or so for several weeks at a time, or there may be exacerbations in which the temperature rises three or four degrees; at this stage, of course, examination of the urine will show the presence of pus.

Investigation of the various deformities of the urinary tract has been simplified since the advent of intravenous pyelography. The examination is one which is easily carried out and with little disturbance to the child, and if there is any reason for suspecting the presence of dilatation or deformity of the kidney pelves and ureters there should be no hesitation in making the investigation.

In conclusion I may mention four cases illustrating rare causes of abdominal pain. The first occurred in a boy aged ten years, who complained of attacks of pain in the right hypochondrium; the attacks were followed by a faint tinge of jaundice, and an enlarged gall-bladder could be felt. Operation disclosed a thickened and distended gall-bladder which was eventually removed, and the child's symptoms then cleared up. The second was a boy who, during convalescence from mumps, developed acute epigastric pain and severe vomiting; his symptoms were due to acute pancreatitis arising as a complication of mumps, and, as usually happens in this condition, a satisfactory recovery was made without operation. The third was a boy who would suddenly stop whatever he was doing, complain for a few seconds of severe abdominal pain, and at the same time pass urine. His attacks were looked upon as a variant of epilepsy, and certainly with the usual treatment for epilepsy his symptoms disappeared. The fourth was an only child who had recently had his appendix removed. On returning to school it was impressed on his schoolmates that he must be treated with gentleness, but they grew tired of doing this and eventually pushed him over and jumped on him. For some weeks after this the patient complained of pain beneath the umbilicus, and he was unable to flex his hips but kept himself rigidly extended like a poker. After a full investigation had failed to show any organic cause for the symptoms he was regarded as hysterical, and after a few minutes of electrical treatment, when it was suggested to him that his pain was being replaced by electricity, his symptoms entirely disappeared !

